# Multimodel inference applied to oxygen recovery kinetics after 6-min walk tests in patients with chronic obstructive pulmonary disease

**DOI:** 10.1371/journal.pone.0187548

**Published:** 2017-11-08

**Authors:** Florent Baty, Christian Ritz, Signe Marie Jensen, Lukas Kern, Michael Tamm, Martin Hugo Brutsche

**Affiliations:** 1 Department of Pulmonary Medicine, Cantonal Hospital St. Gallen, St. Gallen, Switzerland; 2 Department of Nutrition, Exercise and Sports, University of Copenhagen, Copenhagen, Denmark; 3 Department of Plant and Environmental Sciences, University of Copenhagen, Copenhagen, Denmark; 4 Department of Pulmonary Medicine, University Hospital Basel, Basel, Switzerland; National and Kapodistrian University of Athens, GREECE

## Abstract

6-min walk tests (6MWT) are routinely performed in patients with chronic obstructive pulmonary disease (COPD). Oxygen uptake ($\dot{V}{\text{O}}_{2}$) kinetics during 6MWT can be modeled and derived parameters provide indicators of patients’ exercise capacity. Post-exercise $\dot{V}{\text{O}}_{2}$ recovery also provides important parameters of patients’ fitness which has not been extensively investigated in COPD. Several nonlinear regression models with different underlying biological assumptions may be suitable for describing recovery kinetics. Multimodel inference (model averaging) can then be used to capture the uncertainty in considering several models. Our aim was to apply multimodel inference in order to better understand the physiological underpinnings of $\dot{V}{\text{O}}_{2}$ recovery after 6MWT in patients with COPD. 61 patients with COPD (stages 2 to 4) were included in this study. Oxygen kinetics during 6MWT were modeled using nonlinear regression. Three statistical approaches (mixed-effects, meta-analysis and weighted regression) were compared in order to summarize estimates obtained from multiple kinetics. The recovery phase was modeled using 3 distinct equations (log-logistic, Weibull 1 and Weibull 2). Three models were fitted to the set of 61 kinetics. A significant model-averaged difference of 40.39 sec (SE = 17.1) in the time to half decrease of $\dot{V}{\text{O}}_{2}$ level ($T_{1/2}\;\dot{V}{\text{O}}_{2}$) was found between stage 2 and 4 (*p* = 0.0178). In addition, the Weibull 1 model characterized by a steeper decrease at the beginning of the recovery phase showed some improvement of goodness of fit when fitted to the kinetics of patients with stage 2 COPD in comparison with the 2 other models. Multimodel inference was successfully used to model $\dot{V}{\text{O}}_{2}$ recovery after 6MWT in patients with COPD. Significant model-averaged differences in $T_{1/2}\;\dot{V}{\text{O}}_{2}$ were found between moderate and very severe COPD patients. Furthermore, specific patterns of $\dot{V}{\text{O}}_{2}$ recovery could be identified across COPD stages.

## Introduction

In patients with chronic obstructive pulmonary disease (COPD), dyspnea is a frequent respiratory symptom which progressively leads to exercise intolerance. In order to assess the exercise capacity of patients with COPD, 6-min walk tests (6MWT) are commonly performed. This submaximal test is of particular interest as it reflects patients daily life activities.

Oxygen uptake ($\dot{V}{\text{O}}_{2}$) kinetics during 6MWT can be modeled using nonlinear regression and derived parameters provide indicators of patients’ exercise capacity [1]. Established parameters include the oxygen uptake at steady state ($\dot{V}{\text{O}}_{2}ss$) and the mean response time (MRT) corresponding to the time needed for $\dot{V}{\text{O}}_{2}$ to reach 63% of $\dot{V}{\text{O}}_{2}ss$.

The post-exercise $\dot{V}{\text{O}}_{2}$ recovery phase also provides important indicators of physical fitness as shown by Cohen-Solal et al. [2] in patients with chronic heart failure. However, it has not been extensively investigated in patients with COPD. In a recent publication, Bellefleur and colleagues showed that patients with COPD undergoing cardiopulmonary exercise tests have a slower kinetics in the early recovery period compared with healthy individuals and that the quarter-time recovery of oxygen uptake ($T_{1/4}\;\dot{V}O_{2}$) increased with the severity of COPD [3]. Modeling approaches have also been used to characterize the recovery phase of patients with COPD and significant differences in the steepness of the $\dot{V}{\text{O}}_{2}$ recovery and the half-time recovery of oxygen uptake ($T_{1/2}\;\dot{V}O_{2}$) have been found across all disease severity stages [4].

In practice, a series of curves from multiple patients are often collected and various modeling strategies can be used to summarize estimates obtained from multiple experiments. Nonlinear mixed-effects models can be fitted to the entire data set [1]. Mixed-effects models are designed to provide parameter estimates by taking into account within- and between-experiments variability. However fitting nonlinear mixed models is not always straightforward and problems of convergence may occur, especially when considering small data sets and complex models. A second strategy consists in combining estimates obtained by separate univariate model fits. In this situation either a meta-analysis strategy or a simpler weighted regression can be chosen to summarize the estimates [5].

Furthermore, the choice of the model taken to describe the recovery phase plays a critical role. A number of nonlinear regression models with partly different underlying biological assumptions and implied mechanisms may be suitable to describe recovery kinetics. Therefore, there is not always an obvious choice in selecting one model over another. In order to capture the uncertainty in considering several models, multimodel inference (a.k.a. model averaging) can be used [6, 7]. Model averaging combines parameter estimates from several candidate models into one single model-averaged estimate corresponding to the weighted mean of the individual estimates. The weight applied to each individual estimate is related to the goodness of fit of the particular model relative to the other models. Model averaging can be applied to mixed-effects, meta-analysis and weighted regression approaches. On the other hand, when analyzing multiple curves originated from a heterogeneous population (e.g., COPD patients with pathophysiological heterogeneity), one model may better fit data acquired in a particular subgroup of patients. In this situation, model selection tools can be used to compare the goodness of fit of several models in various subgroups of the population.

Our aim was to apply and compare results from multimodel inference for three statistical approaches—mixed-effects, meta-analysis and weighted regression—in order to possibly achieve improved characterizations of the physiological underpinnings of the $\dot{V}{\text{O}}_{2}$ recovery after 6MWT in patients with COPD. Mixed-effects models are based on individual measurements from all patients whereas meta analysis and weighted analysis of variance (ANOVA) are fitted using summary data, i.e., parameter estimates obtained from models fitted separately to data from each patient. We hypothesized that the pattern of $\dot{V}{\text{O}}_{2}$ recovery may differ among the different groups of disease severity, with potential clinical implications on the indidualized handling of patients with COPD before, during and after exercise testing.

## Materials and methods

### COPD data set

We performed a cross-sectional observational study in patients with COPD [8]. Patients referred for a 6MWT at the Department of Pulmonary Medicine of the University Hospital Basel, Switzerland between August 2003 and June 2007 were considered for participation in the study. Exclusion criteria were as follows: need for oxygen supply or resting transcutaneous oxygen saturation (SpO_2_) of < 85% while breathing room air, inability to walk, any acute coronary event during the previous month and conditions precluding the use of a face mask (e.g., anatomic anomaly, claustrophobia or panic disorder).

Patients gave their written informed consent to participate in the study. The study was approved by the local institutional review board (Ethikkommission beider Basel). The data analyzed in the present study were fully anonymized and no individual clinical data are presented. A minimal anonymized supporting data set is provided in S1 Dataset. Further study details can be found in previous publications [8–10].

### Oxygen monitoring during 6MWT

The Oxycon Mobile^®^ (Viasys Healthcare, USA) portable, wireless cardiopulmonary exercise testing device was used to measure breath-by-breath $\dot{V}{\text{O}}_{2}$ consumption. Pulse rate was determined by using an ECG-triggered belt (Polar^®^ Electro OY T-61). Blood oxygen saturation level (SpO_2_) was measured by using a finger clip. $\dot{V}{\text{O}}_{2}$ and carbon dioxide output ($\dot{V}{\text{CO}}_{2}$), tidal volumes and breathing frequency were assessed by using a facemask (dead space < 70 mL) with a flow sensor and a gas analyzer. The patient carried data storage and transfer units by using a dedicated harness. Wireless transfer of breath-by-breath data to a laptop computer allowed real-time monitoring. The additional weight (950 g) of the equipment had no effect on walking distance [8]. The exact 6MWT procedure with mobile telemetry has been previously described [8]. Original breath-by-breath data were imported from the mobile telemetry device. Raw data were pre-processed by averaging the breath-by-breath measurements over consecutive periods of 20 seconds in agreement with the recommendations from the American Thoracic Society on cardiopulmonary exercise testing [11]. Since the optimal averaging period in breath-by-breath data is still debated [12–14], we carried out a sensitivity analysis in order to evaluate the influence of the choice of the averaging period (5-, 10-, and 15-sec) on our findings.

### A set of nonlinear regression models

The recovery phase was modelled using three different equations. One equation describes a symmetrical sigmoid pattern (log-logistic, Eq 1) while the two others depict asymmetrical sigmoid patterns with an inflection point located either at the beginning or at the end of the recovery kinetics (Weibull models 1 and 2, Eqs 2 and 3, respectively).

$$\begin{array}{c} \begin{array}{cc} \dot{V}O_{2}\left(t\right)= & \dot{V}O_{2}rest+\left(\dot{V}O_{2}ss-\dot{V}O_{2}rest\right)\left(1-\exp\left(-\left(t-\lambda \right)/\tau_{1}\right)\right)+\\ & \left(\dot{V}O_{2}rec-\dot{V}O_{2}ss\right)/\left(1+\exp\left(\tau_{2}\times \log\left(\left(t-\left(\lambda_{max}+360\right)\right)/T_{1/2}\;\dot{V}O_{2}\right)\right)\right) \end{array} \end{array}$$ (1)

$$\begin{array}{c} \begin{array}{cc} \dot{V}O_{2}\left(t\right)= & \dot{V}O_{2}rest+\left(\dot{V}O_{2}ss-\dot{V}O_{2}rest\right)\left(1-\exp\left(-\left(t-\lambda \right)/\tau_{1}\right)\right)+\\ & \left(\dot{V}O_{2}rec-\dot{V}O_{2}ss\right)\left(\exp\left(-\exp\left(\tau_{2}\times \log\left(\left(t-\left(\lambda_{max}+360\right)\right)/T_{1/2}\;\dot{V}O_{2}\right)\right)\right)\right) \end{array} \end{array}$$ (2)

$$\begin{array}{c} \begin{array}{cc} \dot{V}O_{2}\left(t\right)= & \dot{V}O_{2}rest+\left(\dot{V}O_{2}ss-\dot{V}O_{2}rest\right)\left(1-\exp\left(-\left(t-\lambda \right)/\tau_{1}\right)\right)+\\ & \left(\dot{V}O_{2}rec-\dot{V}O_{2}ss\right)\left(1-\exp\left(-\exp\left(\tau_{2}\times \log\left(\left(t-\left(\lambda_{max}+360\right)\right)/T_{1/2}\;\dot{V}O_{2}\right)\right)\right)\right) \end{array} \end{array}$$ (3)

with $\dot{V}{\text{O}}_{2}rest$, $\dot{V}{\text{O}}_{2}ss$ and $\dot{V}{\text{O}}_{2}rec$ the oxygen level at rest, steady state during exercise and recovery, respectively; *τ*_1_ the growth rate of the mono-exponential $\dot{V}{\text{O}}_{2}$ function during 6MWT; *τ*_2_ the steepness of the exponential decay during the recovery phase and $T_{1/2}\;\dot{V}O_{2}$ the time for half decrease of the $\dot{V}{\text{O}}_{2}$ level in the recovery phase. λ is the length of the resting period, which is controlled by the experimenter and therefore not estimated during the fitting procedure. The maximum length of the resting period among the experiments (λ_*max*_) is determined *a priori* hence it need not be estimated during the fitting procedure; it is used to “align” multiple kinetics by removing differences in the duration of individual resting phases.

### Mixed effects modeling

In mixed-effects models, individual experiments are treated as samples taken from a population by means of random effects [15].

For *i* = 1, …, *m* patients, the following models were assumed:

$$\begin{array}{c} y_{ij}=\dot{V}O_{2}\left(t_{ij},\beta_{i}\right)+\epsilon_{ij} \end{array}$$

where *y*_*ij*_ are the response vectors of length *j* = 1, …, *n*_*i*_ with the corresponding vectors of individual times *t*_*ij*_. The nonlinear function such as the above six-parameter models (Eqs 1, 2 and 3) evaluated at time *t*_*ij*_ is denoted by $\dot{V}O_{2}\left(t_{ij},\beta_{i}\right)$ with a *p*-dimensional patient-specific parameter *β*_*i*_. The residual vectors $\epsilon_{ij}∼N\left(0,\sigma^{2}\Lambda_{i}\right)$ are assumed to be normally distributed with a correlation structure defined by the elements of the matrices Λ_*i*_; for the current COPD data set we assumed that Λ_*i*_ is the identity matrix. The curve is described by the functions $\dot{V}O_{2}\left(t_{ij},\beta_{i}\right)$ with a patient-specific (*p* × 1) vector of parameters *β*_*i*_.

Between-patient effects are described by modeling the *β*_*i*_. These effects are separated into fixed and random effects:

$$\begin{array}{c} \beta_{i}=A_{i}\beta +B_{i}b_{i} \end{array}$$

where *β* is the vector of fixed-effects parameters and *A*_*i*_ the design matrix of patient characteristics. Differences between patients not captured by the recorded patient characteristics, are described by the patient-specific random effects vector *b*_*i*_; these random effects may possibly be modified through explanatory variables encoded in the corresponding design matrix *B*_*i*_. Random effects are assumed to follow a mean-zero, possibly multivariate normal distribution: $b_{i}∼N\left(0,G\right)$ where *G* denotes the between-patient variance-covariance matrix.

In our example, the nonlinear mixed-effects regression models were parametrized as follows: each individual $\dot{V}{\text{O}}_{2}$ kinetics defines one cluster for which different mean trends for the different disease stages (2 to 4) is assumed (all 6 parameters defined as fixed effects); random effects were specified for the four parameters that characterize the recovery phase $\dot{V}{\text{O}}_{2}ss$, $T_{1/2}\;\dot{V}O_{2}$, *τ*_2_, $\dot{V}{\text{O}}_{2}rec$. In this particular case, *A*_*i*_ is defined as the dummy coded design matrix specifying disease stage specific fixed-effect parameters, and *B*_*i*_ is defined as the random effect design matrix using dummy coded patient identifiers for four of the nonlinear model parameters.

### Meta-analytic approach

The meta-analytic approach is a two-step procedure [5, 16]. In the first step a nonlinear regression model is fitted to data from each patient separately. In the second step the estimate for the parameter of interest is extracted from each of the model fits obtained in the first step. The corresponding standard error is also extracted. A meta analysis may be conducted based on the parameter estimates and standard errors [17]. Specifically, we define ${\hat{\phi }}_{i}$ to be the parameter estimate derived for the *i*^*th*^ patient. Then the meta-analytic random-effects model may be defined as follows:

$$\begin{array}{c} {\hat{\phi }}_{i}=\theta_{i}+A_{i}+\epsilon_{i} \end{array}$$ (4)

where *θ*_*i*_ is the unknown true parameter estimate for the *i*^*th*^ patient (there are only few different *θ*’s corresponding to the categories that the patients are divided into) and $\epsilon_{i}∼N\left(0,\sigma_{i}^{2}\right)$ where *σ*_*i*_ denote the estimated standard error for the *i*^*th*^ patient (from the first step). Note that this means that no residual standard error is estimated from the data. The *A*_*i*_’s are random effects, which are assumed to be normally distributed $N(0,\tau^{2})$ with *τ*^2^ being the heterogeneity variance between patients. The model is commonly fitted using maximum likelihood or restricted maximum likelihood [17].

### Weighted regression approach

A modification of the meta-analytic two-step approach is to assume that the *ϵ*_*i*_’s are distributed as follows: $\epsilon_{i}∼N\left(0,\sigma^{2}\sigma_{i}^{2}\right)$ where the residual standard error *σ* has to be estimated from the data. This analysis is referred to as the weighted regression approach. As a consequence of introducing the residual standard error, estimation has to be carried out using different procedures in statistical softwares; it is strictly speaking no longer a meta analysis but just a weighted regression.

### Model averaging

Model averaging is commonly used to capture uncertainty due to model selection [7]. If one considers *P* candidate models to be fitted to a data set, and *θ* the derived parameter of interest, the model averaged estimate from the *P* models is given by:

$$\begin{array}{c} {\hat{\theta }}_{MA}=\sum_{p=1}^{P}w_{p}{\hat{\theta }}_{p} \end{array}$$

where *w*_*p*_s are model-specific weights $\left(\sum_{p=1}^{P}w_{p}=1\right)$ defined as $w_{p}=\exp\left(-\Delta_{p}/2\right)/(\sum_{r=1}^{P}\exp\left(-\Delta_{r}/2\right)$), with Δ_*p*_ = *IC*_*p*_ − *IC*_min_ and *IC*_*p*_ being the information criterion evaluated for the model *p* ($IC_{\min}=\min_{p=1}^{P}IC_{p}$). An conservative approximation of the unconditional variance of the estimate [18] is given by:

$$\text{var}\left({\hat{\theta }}_{MA}\right)={\left(\overset{P}{\underset{p=1}{\sum }}w_{p}\sqrt{\hat{\text{var}}\left({\hat{\theta }}_{p}\right)+{\left({\hat{\theta }}_{p}-{\hat{\theta }}_{MA}\right)}^{2}}\right)}^{2}$$

### Model selection

The Akaike’s information criterion (AIC) was used for model selection. AIC is defined as:

$$\begin{array}{c} \text{AIC}=-2(\text{log}-\text{likelihood})+2K \end{array}$$

where *K* is the number of estimated parameters included in the model.

AIC provides for a given data set (or subset) a measure of the strength of evidence for plausible biological assumptions / mechanisms associated with a given model relative to a set of other models considered [19]. The model with the lowest AIC is the best model among all models for a given data set.

### Software

All analyses were done using the R statistical software (version 3.3.2) [20]. Nonlinear mixed effects modeling was performed using the package medrc [1, 21] which combines functionalities of the packages drc [22] (nonlinear regression) and nlme [23] (mixed effects modeling). The package metafor was used for meta-analysis [17] and multcomp for statistical inference [24]. Packages AICcmodavg [19] and MuMIn [6] were used for multimodel inference (model averaging) and model selection.

## Results

### Modeling $\dot{V}{\text{O}}_{2}$ recovery kinetics using three summarization strategies

Oxygen kinetics were measured in 61 patients with COPD (Global Initiative for Chronic Obstructive Lung Disease (GOLD) stages 2, 3 and 4). Patients characteristics and anthropometrics are presented in Table 1.

**Table 1:** Anthropometrics, pulmonary functions, cardio-pulmonary exercise capacity. Values are presented as median [IQR].

	COPD GOLD stage		
	2	3	4
**Anthropometrics**			
Subjects, n	21	30	10
Female/male	10/11	10/20	5/5
Age, yr	72.0 [59.0-77.0]	67.5 [61.0-71.0]	60.5 [52-62]
BMI (kg/m^2^)	28.1 [25.5-32.0]	24.3 [21.8-28.0]	20.0 [18.8-20.6]
**Pulmonary functions**			
FEV_1_, L	1.6 [1.3-1.8]	1.0 [0.8-1.1]	0.7 [0.7-0.8]
FEV_1_, % predicted	59.0 [58.0-66.0]	36.5 [34.0-42.0]	26.5 [26.0-28.0]
FEV_1_/FVC, ratio	0.6 [0.5-0.6]	0.4 [0.3-0.5]	0.4 [0.3-0.4]
**Exercise capacity**			
6MWD, m	370.0 [300.0-438.0]	352.5 [290.0-392.0]	345.0 [265.0-374.0]

BMI: body mass index; FEV_1_: forced expiratory volume in 1 sec; FEV_1_ / FVC ratio: forced expiratory volume in 1 sec (FEV_1_) expressed as percent of the forced vital capacity (FVC); 6MWD: 6-minute walking distance.

**Fig 1:**
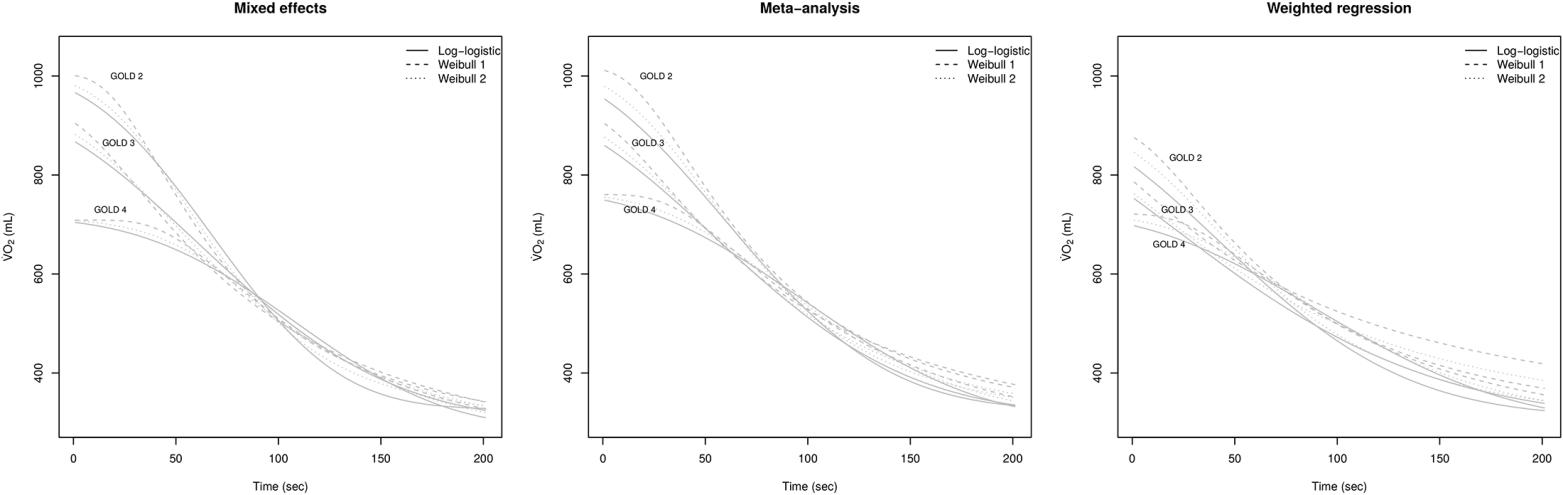
Fitted curves of three models on oxygen kinetics recovery summarized within each COPD disease stage (2-4) for the mixed-effects, meta-analysis and weighted regression approach (left, central and right panels, respectively). The log-logistic, Weibull 1 and Weibull 2 models are displayed by plain, dashed and dotted lines, respectively.

Three approaches were used to summarize the fit of three distinct models on a data set including 61 $\dot{V}{\text{O}}_{2}$ kinetics. Fig 1 shows the recovery kinetics estimated by the mixed effects, meta-analysis and weighted regression strategies (left, central and right panels, respectively). Within each strategy, recovery curves are summarized for each of the three models and for all of the three COPD disease severity stages (GOLD stages 2, 3 and 4). The $T_{1/2}\;\dot{V}{\text{O}}_{2}$ estimates obtained for each model within each of the 3 statistical approaches are summarized in Table 2. Due to their asymmetrical shapes, Weibull models provide either lower (Weibull 1) or higher (Weibull 2) $T_{1/2}\;\dot{V}{\text{O}}_{2}$ estimates than the symmetrical log-logistic model. Independent of choice of statistical approach and model, a significant increase of about 40 seconds in $T_{1/2}\;\dot{V}{\text{O}}_{2}$ was observed between patients with moderate and severe COPD (stages 2 vs. 4). Both the mixed effects and meta-analysis strategies resulted in very similar $T_{1/2}\;\dot{V}{\text{O}}_{2}$ estimates, whereas weighted regression provided estimates which tended to shrink towards the overall mean. Moreover, the choice of the breath-by-breath averaging period did not impact significantly on the $T_{1/2}\;\dot{V}{\text{O}}_{2}$ estimates (S1 Table).

**Table 2:** Time for half-decrease of the $\dot{V}O_{2}$ level during recovery ($T_{1/2}\;\dot{V}O_{2}$) parameter estimates obtained using 3 models and 3 statistical approaches. The estimates are provided together with their associated standard error (SE) and *p*-values. The difference in $T_{1/2}\;\dot{V}O_{2}$ between patients with moderate and severe COPD (GOLD stages 4 vs. 2) is also reported.Three summarization methods are investigated (mixed effects, meta-analysis and weighted regression), and three nonlinear regression models (log-logistic, Weibull 1, and Weibull 2) are compared.

			$T_{1/2}\;\dot{V}O_{2}$		
Method	Model	Disease stage	Estimate	SE	*p*-value
Mixed effects	Log-logistic	COPD 2	127.79	9.22	< 0.001
		COPD 3	133.12	8.05	< 0.001
		COPD 4	168.20	14.40	< 0.001
		COPD (4-2)	40.40	17.05	0.018
	Weibull 1	COPD 2	112.76	9.42	< 0.001
		COPD 3	113.90	8.15	< 0.001
		COPD 4	150.94	14.62	< 0.001
		COPD (4-2)	38.17	17.34	0.028
	Weibull 2	COPD 2	144.51	8.87	< 0.001
		COPD 3	154.06	7.79	< 0.001
		COPD 4	187.37	13.89	< 0.001
		COPD (4-2)	42.86	16.43	0.009
Meta-analysis	Log-logistic	COPD 2	128.03	8.53	< 0.001
		COPD 3	130.88	7.51	< 0.001
		COPD 4	164.31	12.47	< 0.001
		COPD (4-2)	36.28	15.11	0.016
	Weibull 1	COPD 2	114.06	8.61	< 0.001
		COPD 3	112.18	7.50	< 0.001
		COPD 4	146.41	12.83	< 0.001
		COPD (4-2)	32.35	15.45	0.036
	Weibull 2	COPD 2	145.24	8.66	< 0.001
		COPD 3	149.39	7.54	< 0.001
		COPD 4	183.55	12.89	< 0.001
		COPD (4-2)	38.31	15.53	0.014
Weighted regression	Log-logistic	COPD 2	121.70	6.18	< 0.001
		COPD 3	121.96	6.23	< 0.001
		COPD 4	159.92	12.75	< 0.001
		COPD (4-2)	38.23	14.17	0.009
	Weibull 1	COPD 2	107.66	6.17	< 0.001
		COPD 3	103.29	6.19	< 0.001
		COPD 4	138.65	13.03	< 0.001
		COPD (4-2)	30.98	14.42	0.036
	Weibull 2	COPD 2	137.33	6.60	< 0.001
		COPD 3	141.49	6.21	< 0.001
		COPD 4	180.95	12.87	< 0.001
		COPD (4-2)	43.62	14.46	0.004

### Multimodel inference / Model averaging

The goodness of fit of the three models for each summarization strategy is reported in Table 3. The model-averaged estimates of the difference of $T_{1/2}\;\dot{V}{\text{O}}_{2}$ ($\Delta T_{1/2}\;\dot{V}O_{2}$) between patients with moderate and severe COPD was calculated within each statistical approach. The mixed-effects strategy provided the largest model-averaged estimates ($\Delta T_{1/2}\;\dot{V}O_{2}=40.4$, SE = 17.1). Both the meta-analysis and weighted regression strategy provided smaller model-averaged estimates of $\Delta T_{1/2}\;\dot{V}O_{2}$: 34.9 (SE = 21.2) and 34.2 (SE = 14.2), respectively.

**Table 3:** Multimodel inference / Model averaging. Akaike information criterion (AIC) and Akaike weights are reported for the 3 models (log-logistic, Weibull 1 and Weibull 2) analyzed through the 3 summarization strategies (mixed effects, meta-analysis and weighted regression). The estimates and model averaged estimates of the difference of the time for half-decrease of the $\dot{V}O_{2}$ level during recovery $T_{1/2}\;\dot{V}O_{2}$ between patients with moderate and severe COPD (GOLD 4 vs. 2) are provided together with their associated standard error (SE).

				$\Delta T_{1/2}\;\dot{V}O_{2}$ (COPD 4—2)	
Method	Model	AIC	Weight	Estimate	SE
Mixed effects	Log-logistic	36556	0.996	40.40	17.05
	Weibull 1	36578	0	38.17	17.34
	Weibull 2	36567	0.004	42.86	16.43
	Model-averaged	-	-	**40.41**	**17.07**
Meta-analysis	Log-logistic	634	0.341	36.28	15.11
	Weibull 1	633	0.449	32.35	15.45
	Weibull 2	634	0.211	38.31	15.53
	Model-averaged	-	-	**34.94**	**21.20**
Weighted regression	Log-logistic	644	0.024	38.23	14.17
	Weibull 1	637	0.737	30.98	14.42
	Weibull 2	639	0.239	43.62	14.46
	Model-averaged	-	-	**34.17**	**14.25**

No statistically significant difference in $\Delta T_{1/2}\;\dot{V}O_{2}$ was found between COPD GOLD 3 and 2, whereas a statistically significant difference was found between COPD GOLD 4 and 3 (S2 Table).

### Model selection in subgroups of patients

The AIC obtained for the fit of each of the three models within each statistical approach and all subgroups defined by the disease severity (COPD stages) were compared. The mixed models approach applied to subsets of data resulted in problems of convergence due to small sample size.

Results obtained from both meta-analysis and weighted regression approaches show that the Weibull 1 model characterized by a steeper decrease at the beginning of the recovery phase showed some improvement of goodness of fit when fitted to the kinetics of the stage 2 patients in comparison with the 2 other models (meta-analysis: AIC = 221.9 in Weibull 1 vs. 223.4 and 222.6 for the log-logistic and Weibull 2 models; weighted regression: AIC = 219.3 in Weibull 1 vs. 220.9 and 219.6 for the log-logistic and Weibull 2).

## Discussion

As shown in the current example from pulmonary medicine, the simultaneous analysis of multiple experiments using a set of plausible models is associated with two important challenges: i) the choice of the statistical approach needed to combine the information from multiple experiments, and ii) the multimodel inference.

We found some differences among statistical approaches. Estimates provided by the mixed-effects and meta-analysis approaches were similar whereas the weighted regression strategy differed more importantly from the 2 other strategies. The mixed effects strategy resulted in estimates showing larger between group differences whereas estimates obtained from the meta-analysis and more importantly the weighted regression tended to shrink towards the overall mean in the data set. In another context (economical science), Stanley and colleagues [25] provided a comparison of weighted regression with random-effects meta-analysis, demonstrating the superiority of the former in comparison to the latter in settings with high heterogeneity and in particular for small sample sizes. Their findings support the results presented here, where we found estimates from the meta-analysis and the weighted regression to differ the most for the stratified analyses containing less observations. On the contrary, heterogeneity between patients may be expected, but to a smaller extend within subgroups of patients with the same disease severity rather than across all patients. It should also be noted that an extra layer of complexity was added here in terms of the multimodel inference and that coverage of the model-averaged estimates is expected to be higher than the nominal level due to the choice of a conservative variance estimates [18].

We successfully applied multimodel inference in our data in order to take into account the uncertainty due to the model selection, resulting in more precise and robust estimates. Although inference was based on model-averaging we used model selection in order to test the plausibility of biological hypotheses underlying different models and get a better idea of the nature of the patterns of oxygen recovery in subgroups of the population of patients with COPD. Independently from the choice of the statistical approach, the goodness of fit of the 3 models within each subgroup of patients was comparable. However, small but consistent improvements of goodness-of-fit were found when fitting the Weibull 1 model to the subgroup of patients with stage 2 COPD in comparison with the 2 other models (log-logistic and Weibull 2). Differences occuring in the early part of the oxygen recovery and possibly originated from distinct physiological processes seem to play a critical role among patients with COPD resulting into pattern variations among the 3 investigated severity stages. This is in line with the findings from Bellefleur and colleagues [3] who showed some significant between-severity stages differences in the early period of the recovery phase.

Physiological mechanisms explaining variations in the speed and pattern of post-exercise recovery phases associated with COPD severity can tentatively be explained. After aerobic exercise, recovery phases are needed to normalize the excess post-exercise oxygen consumption (EPOC) [26]. Excess oxygen is required to rebuild adenosine triphosphatase and phosphocreatine [27, 28], and is also involved in the removal of accumulated lactic acid. Prolonged recovery kinetics observed in more severe patients with COPD might be attributed to slow respiratory gas exchanges [29], or slow recovery of energy stores in peripheral skeletal muscles [30]. The present study shows that some of the physiological recovery mechanisms occurring immediately after exercise are more efficiently initiated in patients with moderate COPD (in comparison with very severe COPD). This may explain the more abrupt oxygen decline observed in the early recovery phase of patients with moderate COPD.

## Conclusion

Multimodel inference is a powerful tool to summarize information from multiple recovery kinetics when modelled by a set of plausible models. Significant model-averaged differences in $T_{1/2}\;\dot{V}O_{2}$ were found between moderate and very severe COPD patients. Furthermore, the pattern of $\dot{V}{\text{O}}_{2}$ recovery differed among COPD stages, patients with moderate COPD showing a steeper decline of their consumption at the beginning of recovery. Finally, our study indicates that recovery kinetics include clinically relevant information about the exercise capacity of patients with COPD which can be apprehended using advanced methodology. In clinical practice, exercise testing protocols should further emphasize the importance of recovery phases, whereas individualized handling of patients based on their disease severity should be advised.

## Supporting information

S1 TableSensitivity analysis of the breath-by-breath averaging pre-processing approach.The time for half-decrease of the $\dot{V}{\text{O}}_{2}$ level during recovery ($T_{1/2}\;\dot{V}O_{2}$) was estimated based on the mixed effects statistical approach using three different time-averaging period (5-sec, 10-sec and 15 sec).(PDF)

S2 TableMultimodel inference / Model averaging.Akaike information criterion (AIC) and Akaike weights are reported for the 3 models (log-logistic, Weibull 1 and Weibull 2) analyzed through the 3 summarization strategies (mixed effects, meta-analysis and weighted regression). The estimates and model averaged estimates of the difference of the time for half-decrease of the $\dot{V}O_{2}$ level during recovery $T_{1/2}\;\dot{V}O_{2}$ between patients with COPD GOLD 3 vs. 2 and GOLD 4 vs. 3 are provided together with their associated standard error (SE).(PDF)

S1 Dataset Supporting oxygen recovery kinetics data set. (RDA)
