# Supplementary material for: Multimodel inference applied to oxygen recovery kinetics after 6-min walk tests in patients with chronic obstructive pulmonary disease
Source: PLoS One. 2017 Nov 8;12(11):e0187548. doi: 10.1371/journal.pone.0187548 (PMC5678891; doi:10.1371/journal.pone.0187548)
Supplement: S1 Table — The time for half-decrease of the V˙O2 level during recovery (T1/2V˙O2) was estimated based on the mixed effects statistical approach using three different time-averaging period (5-sec, 10-sec and 15 sec). (PDF) [file pone.0187548.s001.pdf]

S1 Table: Sensitivity analysis of the breath-by-breath averaging pre-processing approach. The time for half-decrease of the $\dot{V}O_2$ level during recovery ($T_{1/2}\dot{V}O_2$) was estimated based on the mixed effects statistical approach using three different time-averaging period (5-sec, 10-sec and 15 sec).

Averaging period	Model	Disease stage	$T_{1/2}\dot{V}O_2$ Estimate	SE	p-value
5-sec	Log-logistic	COPD 2	135.66	9.67	< 0.001
		COPD 3	138.91	8.21	< 0.001
		COPD 4	175.90	14.50	< 0.001
		COPD (4-2)	40.24	17.42	0.021
	Weibull 1	COPD 2	120.94	9.72	< 0.001
		COPD 3	120.26	8.24	< 0.001
		COPD 4	158.29	14.59	< 0.001
		COPD (4-2)	37.36	17.51	0.033
	Weibull 2	COPD 2	152.36	9.52	< 0.001
		COPD 3	158.01	8.09	< 0.001
		COPD 4	194.33	14.27	< 0.001
		COPD (4-2)	41.96	17.14	0.014
10-sec	Log-logistic	COPD 2	132.30	9.49	< 0.001
		COPD 3	137.10	8.18	< 0.001
		COPD 4	172.88	14.54	< 0.001
		COPD (4-2)	40.58	17.33	0.019
	Weibull 1	COPD 2	117.87	9.67	< 0.001
		COPD 3	118.28	8.26	< 0.001
		COPD 4	155.08	14.68	< 0.001
		COPD (4-2)	37.21	17.55	0.034
	Weibull 2	COPD 2	149.76	9.60	< 0.001
		COPD 3	155.87	8.21	< 0.001
		COPD 4	191.73	14.60	< 0.001
		COPD (4-2)	41.97	17.45	0.014
15-sec	Log-logistic	COPD 2	130.25	9.26	< 0.001
		COPD 3	135.57	8.04	< 0.001
		COPD 4	170.04	14.26	< 0.001
		COPD (4-2)	39.79	16.96	0.019
	Weibull 1	COPD 2	115.49	9.49	< 0.001
		COPD 3	117.02	8.19	< 0.001
		COPD 4	152.56	14.49	< 0.001
		COPD (4-2)	37.08	17.28	0.032
	Weibull 2	COPD 2	146.78	8.79	< 0.001
		COPD 3	156.23	7.66	< 0.001
		COPD 4	188.71	13.57	< 0.001
		COPD (4-2)	41.93	16.13	0.009
