# Supplementary material for: Multimodel inference applied to oxygen recovery kinetics after 6-min walk tests in patients with chronic obstructive pulmonary disease
Source: PLoS One. 2017 Nov 8;12(11):e0187548. doi: 10.1371/journal.pone.0187548 (PMC5678891; doi:10.1371/journal.pone.0187548)
Supplement: S2 Table — Akaike information criterion (AIC) and Akaike weights are reported for the 3 models (log-logistic, Weibull 1 and Weibull 2) analyzed through the 3 summarization strategies (mixed effects, meta-analysis and weighted regression). The estimates and model averaged estimates of the difference of the time for half-decrease of the V˙O2 level during recovery T1/2V˙O2 between patients with COPD GOLD 3 vs. 2 and GOLD 4 vs. 3 are provided together with their associated standard error (SE). (PDF) [file pone.0187548.s002.pdf]

S2 Table: Multimodel inference / model averaging. Akaike information criterion (AIC) and Akaike weights are reported for the 3 models (log-logistic, Weibull 1 and Weibull 2) analyzed through the 3 summarization strategies (mixed effects, meta-analysis and weighted regression). The estimates and model averaged estimates of the difference of the time for half-decrease of the $\dot{V}O_2$ level during recovery $T_{1/2}\dot{V}O_2$ between patients with COPD GOLD 3 vs. 2 and GOLD 4 vs. 3 are provided together with their associated standard error (SE).

				$\Delta T_{1/2}\dot{V}O_2$ (COPD 3 - 2)		$\Delta T_{1/2}\dot{V}O_2$ (COPD 4 - 3)	
Method	Model	AIC	Weight	Estimate	SE	Estimate	SE
Mixed effects	Log-logistic	36556	0.996	5.33	12.21	35.07	16.45
	Weibull 1	36578	0	1.14	12.42	37.03	16.69
	Weibull 2	36567	0.004	9.55	11.77	33.31	15.88
	Model-averaged	-	-	**5.35**	**12.26**	**35.07**	**16.46**
Meta-analysis	Log-logistic	634	0.341	2.85	11.36	33.43	14.56
	Weibull 1	633	0.449	-1.88	11.41	34.23	14.86
	Weibull 2	634	0.211	4.15	11.49	34.15	14.93
	Model-averaged	-	-	**1.00**	**18.29**	**34.94**	**14.91**
Weighted regression	Log-logistic	644	0.024	0.26	8.77	37.96	14.19
	Weibull 1	637	0.737	-4.37	8.74	35.36	14.42
	Weibull 2	639	0.239	-4.16	9.06	39.47	14.29
	Model-averaged	-	-	**-2.23**	**8.67**	**36.40**	**14.16**
